# Final HIV status outcome for HIV-exposed infants at 18 months of age in nine states and the Federal Capital Territory, Nigeria

**DOI:** 10.1371/journal.pone.0263921

**Published:** 2022-02-14

**Authors:** Babatunde Adelekan, Bidemi Harry-Erin, Martha Okposo, Ahmad Aliyu, Nicaise Ndembi, Patrick Dakum, Nadia A. Sam-Agudu

**Affiliations:** 1 Strategic Information, Institute of Human Virology Nigeria, Abuja, Nigeria; 2 Laboratory Research, Institute of Human Virology Nigeria, Abuja, Nigeria; 3 Department of Pediatrics, Institute of Human Virology, University of Maryland School of Medicine, Baltimore, Maryland, United States of America; 4 Prevention, Care and Treatment Unit, Institute of Human Virology Nigeria, Abuja, Nigeria; 5 International Research Center of Excellence, Institute of Human Virology Nigeria, Abuja, Nigeria; University of Ghana College of Health Sciences, GHANA

## Abstract

**Introduction:**

While antiretroviral therapy (ART) coverage for pregnant women has undergone steady scale-up, Nigeria’s final mother- to-child transmission of HIV (MTCT) rate remains unacceptably high at 10%. This study aimed to determine final outcomes (MTCT rates) and their correlates among HIV-exposed infants (HEI) in nine states and the Federal Capital Territory, Nigeria.

**Methods:**

This retrospective, cross-sectional study was conducted at 96 primary, secondary and tertiary health facilities supported by the Institute of Human Virology Nigeria. Data was abstracted for a birth cohort of HEI born between October 30, 2014 and April 30, 2015 whose 18–24 month final outcome was assessed by October 30, 2016. Only infants with a six-week first DNA PCR result, and a rapid HIV antibody test result at age 18 to 24 months were included. Multivariate logistic regression (adjusted odds ratios [aORs]) evaluated for predictors of HIV positivity at ≥18 months.

**Results:**

After testing at ≥18 months, 68 (2.8%) of the 2,405 exposed infants in the birth cohort were HIV-positive. After a minimum of 18 months of follow-up, 51 (75%) HIV-positive infants were alive on ART; 7 (10%) had died, 5 (7.3%) were lost to follow-up and 5 (7.3%) were transferred out. Rural maternal residence, lack of maternal ART/ARV prophylaxis, mixed infant feeding and infant birth weight less than 2.5 kg correlated with an HIV-positive status for infant final outcomes.

**Conclusion:**

The final HIV positivity rate of 2.8% is encouraging, but is not population-based. Nevertheless, supported by our findings, we recommend continued programmatic focus on early access to quality prenatal care and maternal ART for pregnant women, especially for women living with HIV in rural areas. Furthermore, implementation of nationwide sensitization and education on six-months’ exclusive infant breastfeeding with concurrent maternal ART should be strengthened and sustained to reduce MTCT rates.

## Introduction

Nigeria has the third -largest HIV burden in the world, after Mozambique and South Africa. In 2020, there were an estimated 21,000 new child HIV infections in Nigeria, the highest in the world [1], which accounted for 14% of the global estimate [2]. Furthermore, at 15% and 10% respectively, Nigeria had the second-highest six-week and breastfeeding mother-to-child transmission of HIV (MTCT) rate in 2020 [1]. The UNAIDS has targeted final transmission rates of <2% and <5% among non-breastfeeding and breastfeeding infants, respectively for the elimination of mother-to-child transmission of HIV [3]. To reduce these high MTCT rates and new child HIV infections, uptake of, and compliance with interventional services in the prevention of mother- to child transmission of HIV (PMTCT) cascade are critically important. The PMTCT cascade is a comprehensive multistep continuum of care to be completed by HIV-positive mother-exposed infant pairs, and includes maternal antenatal HIV testing and treatment, antenatal and delivery care, early infant diagnosis, postnatal services and linkage to long-term HIV care and support, which includes final HIV outcome testing for the HEI at 18 months of age [4].

From its inception to date, the national PMTCT program has been coordinated by the Federal Government of Nigeria and implemented largely at the subnational level by states, with substantial donor contributions such as from the US President’s Emergency Plan for AIDS Relief (PEPFAR). Federally-issued national guidelines drive PMTCT service implementation in line with scientific developments and international best practices based on World Health Organization (WHO) recommendations, with the overall goal to improve maternal health and child survival through accelerated provision of comprehensive and integrated PMTCT services. The Nigerian PMTCT program started in tertiary institutions in 2002 and was later decentralized to secondary hospitals and primary healthcare centers in both public and private settings. Technical, financial and logistic support is provided to PMTCT programs at both public and private facilities by donors and partners, including PEPFAR and its non-governmental organization implementing partners [5].

Along with reporting encouraging strides in the global elimination of MTCT agenda, *The Global Plan towards the Elimination of New HIV Infections among Children by 2015 and Keeping Their Mothers Alive* [6] highlighted Nigeria’s wide PMTCT cascade performance gaps. This included low maternal antiretroviral therapy (ART) coverage (30%) and poor early infant diagnosis testing rates (11%), coupled with high rates of early and late MTCT. Ultimately, only 21% of HIV-infected Nigerian children received ART at the end of the Global Plan’s 2011 to 2015 assessment period [3]. Additionally, a 2008 to 2014 audit from the Nigerian national HIV program reported only limited improvements in antenatal maternal HIV testing, with up to 42% of HIV-positive women at PMTCT facilities not receiving ART [7]. The audit also reported maternal ART coverage at only 30% in 2014 [7], however it did not provide PMTCT cascade information on any infant nor maternal outcomes beyond antenatal ART coverage. More recently, in 2020, only 32% of pregnant women in Nigeria received HIV testing, and 44% accessed maternal ART [1]. Also, only 27% of HIV-exposed infants (HEI) received a virological test for HIV within 2 months of birth [8], with a cumulative final MTCT rate of 25% in this group [1, 8].

There is published data on the early part of the PMTCT cascade in Nigeria, largely antenatal maternal outcomes through early infant diagnosis at 2 months [7–13]. However, there is little available data on the latter part of the cascade, particularly on final HIV status outcomes for HEI at or after 18 months of age [14, 15]. Besides generating more data on HEI final outcomes, it is important to understand the factors that potentiate or prevent HIV-infection in this population. This study aimed to determine final outcomes (MTCT rates) and their correlates among HIV-exposed infants in nine states and the Federal Capital Territory in Nigeria, a country critical to achieving the global goal for elimination of mother-to-child transmission of HIV.

## Methods

### Study setting

This retrospective cross-sectional study focused on HEI seen at public and private healthcare facilities across nine states and the Federal Capital Territory in Nigeria, namely, Benue, Delta, Ekiti, Kano, Katsina, Nasarawa, Ogun, Ondo and Osun. HIV services at healthcare facilities in these states were supported by the Institute of Human Virology Nigeria (IHVN) with PEPFAR funding. IHVN is a large local non-governmental organization that provides public health services including for malaria, tuberculosis, and HIV, to healthcare facilities in Nigeria [16].

The primary, secondary and tertiary facilities supported by IHVN and included in the analysis were located across the spectrum of rural and urban settings, with primary facilities located largely in rural areas, secondary facilities in both rural and urban areas, and tertiary facilities concentrated in urban areas. Additionally, tertiary facilities also serve as referral centers at which patients from within and outside the state access higher-level care.

### Study population and data collection

We reviewed available records of infants born between October 30, 2014 and April 30, 2015 who had final outcome results available by October 30, 2016. This birth cohort attained 18 to 24 months of age by October 30, 2016. We prioritized data analysis for this birth cohort of HEI because 2014 was the first year of implementation for a newly-implemented longitudinal birth cohort register that linked the mother-infant pair in the PMTCT program. HIV positive final outcome refers to the status of HEIs identified as HIV-infected after testing positive for HIV antibodies at ≥18 months of age and/or 6 weeks after cessation of breastfeeding [17].

In 2014/2015, national guidelines recommended that all HEI were to be tested for HIV by DNA PCR using dried blood samples, for early infant diagnosis (EID) between 6 weeks and 2 months of age, and at 6 weeks after breastfeeding [17]. Infants older than 9 months could first be screened with rapid antibody testing, and if positive, undergo PCR testing for confirmation [17]. There was no birth testing nor mandatory repeat PCR testing recommendation after first DNA PCR for HEI in 2014/2015 [17]. HEI testing negative at first DNA PCR were tested for final outcome by rapid antibody testing between 18 and 24 months of age [17, 18]. The delay period of final outcome testing to up to 24 months is to reduce the possibility of false positives from maternal antibodies which may occur in younger children.

Only HEI with an available DNA PCR result at ≤6 weeks of birth and an HIV rapid test result at ≥18 months of age were eligible for inclusion. All HEI who had a positive DNA PCR test collected between 6 weeks and less than 18 months of age were excluded from final outcome analysis. In addition to HEI HIV test data, socio-demographic and clinical parameters were collected on infants and their HIV-positive mothers. Site-level data clerks de-identified data from IHVN’s electronic HIV program database and from hard-copy program registers at supported facilities in the nine states and the Federal Capital Territory. State-level Monitoring and Evaluation program officers collated site data and submitted it to the Central Strategic Information Unit in secured, encrypted Excel files for analysis.

### Statistical analysis

After abstraction and cleaning, relevant data from mother-infant pairs meeting the eligibility criteria were migrated into Stata 14 (StataCorp. 2015. *Stata Statistical Software*: *Release 14*. College Station, TX: StataCorp LP). Descriptive analysis was performed, after which bi-variate analysis using Chi-square, Fisher’s exact and likelihood ratios were conducted to test for associations between HEI final outcomes and maternal-infant characteristics. Explanatory variables significant at p<0.05 from bi-variate analysis were inputted into a model for multivariate logistic regression to establish predictors of HIV infant positivity at 18–24 months of age. The binary dependent variable was HEI who were positive or negative after HIV rapid testing at ≥18 months. Independent variables were divided into two main domains: maternal data including age and marital, education and employment status at antenatal care clinic booking, facility type attended, place of delivery, and receipt of ART; and infant data including gender, birth weight, DNA PCR result, and infant feeding practice.

### Ethical approval

The study was approved by the Nigerian National Health Research Ethics Committee (NHREC/01/01/2007-01/03/2021D) for secondary analysis of routine data collected by the Institute of Human Virology Nigeria’s HIV program. The data reviewed and presented in this study were collected as part of routine HIV program activities.

## Results

Data were collected from 96 IHVN-supported healthcare facilities (19 private, 77 public) across nine states and the Federal Capital Territory in Nigeria. Data from 2,405 HEI and their HIV-infected mothers were included in our analysis (Table 1). Of the 96 study facilities, 55 (57.3%) were primary-level, 28 (29.2%) secondary and 13 (13.5%) tertiary. Approximately 50% of the mother-infant pairs in our cohort received PMTCT services at tertiary-level facilities.

**Table 1:** Distribution of study participants and facility type.

Facility Level Type n (%)	Number of HEI n (%)
Primary: 55 (57.3)	481 (20.0)
Secondary: 28 (29.2)	721 (30.0)
Tertiary: 13 (13.5)	1,203 (50.0)
**Total: 96**	**Total: 2,405**

### Socio-demographic and clinical characteristics of mother-infant pairs

Approximately 94% of the pregnant women living with HIV in this analysis were married, 63% had at least a primary education while 56% were employed. Almost all (97%) of the women in this cohort delivered at a health facility and via spontaneous vaginal delivery. Our study population of HEI comprised 52.7% (1,257) males (Table 2) and had a mean (±SD) birth weight of 2.96 (±0.465) kg.

**Table 2:** Socio-demographic and clinical characteristics of mother-infant pairs.

	Final HIV Status Outcome		**Total HEI (N) = 2405 n (%)	P value
	*HIV positive (N = 68) n (%)	*HIV negative (N = 2,337) n (%)		
**Maternal** ^a^ **-Infant characteristics**				
**Marital status**				
Married	68 (3.0)	2,195 (97.0)	2,263 (94.1)	0.032^g^
Not Married^b^	0 (0.0)	142 (100.0)	142 (5.9)	
**Maternal Level of Education**				
No education	0 (0.0)	59 (100.0)	59 (2.5)	0.007^g^
Primary	15 (3.1)	463 (96.9)	478 (19.9)	
Secondary/Post-Secondary	19 (1.8)	1,020 (99.2)	1,039 (43.2)	
Unknown	34 (4.1)	795 (95.9)	829 (34.5)	
**Maternal Occupation**				
Working	46 (3.4)	1,290 (96.6)	1,336 (55.6)	0.042
Not working	22 (2.1)	1,047 (97.9)	1,069 (44.6)	
**Maternal Residence**				
Rural	23 (4.3)	516 (95.7)	539 (22.4)	0.002^f^
Urban	35 (2.1)	1,666 (97.2)	1,701 (70.7)	
Unknown	10 (6.1)	155 (93.9)	165 (6.5)	
**Maternal Age (Years)**				
<25	9 (3.07)	284 (96.9)	293 (12.2)	0.727^f^
25–49	59 (2.8)	2,043 (97.2)	2,102 (87.4)	
50+	0 (0.0)	10 (100.0)	10 (0.4)	
**Maternal place of delivery**				
Health Facility	57 (2.6)	2,107 (97.4)	2,164 (90.0)	0.097
Non-health facility^c^	10 (5.6)	173 (94.4)	178 (7.4)	
Unknown	1 (1.6)	62 (98.4)	63 (2.6)	
**Maternal Mode of delivery**				
Spontaneous Vaginal	66 (2.9)	2,178 (97.1)	2,244 (93.3)	0.321^g^
Caesarean section	2 (1.2)	159 (98.8)	161 (6.7)	
**Maternal ANC CD4** ^h^				
<500	7 (1.3)	526 (98.7)	533 (22.2)	0.004
500+	6 (1.5)	389 (98.5)	395 (16.4)	
Unknown	55 (3.7)	1,422 (96.3)	1,477 (61.4)	
**Maternal ART/ARV Prophylaxis** ^d^				
Yes	45 (2.0)	2,157 (98.0)	2,202 (91.6)	<0.001
No	4 (16.7)	20 (83.3)	24 (1.0)	
Unknown	19 (10.6)	160 (89.4)	179 (7.4)	
**Infant Characteristics and Information**				
**Infant Weight (Kg)**				
<2.5	13 (5.1)	240 (94.9)	252 (10.5)	0.019
2.5+	55 (2.6)	2,097 (97.4)	2,152 (89.5)	
**Infant sex**				
Male	35 (2.8)	1,232 (97.2)	1,267 (52.7)	
Female	33 (2.9)	1,105 (97.1)	1,138 (47.3)	0.839
**Infant DNA PCR result** ^e^				
Positive	60 (90.9)	6 (9.1)	66 (2.7)	<0.001
Negative	8 (0.3)	2,325 (99.7)	2,333 (97.0)	
Unknown	0 (0.0)	6 (100.0)	6 (0.3)	
**Infant feeding practice**				
Mixed	12 (27.9)	31 (72.1)	43 (1.8)	<0.001^f^
Exclusive Breastfeeding	51 (2.3)	2,126 (97.7)	2,177 (90.5)	
Exclusive Replacement	0 (0.0)	100 (100.0)	100 (4.2)	
Unknown	5 (5.9)	80 (94.1)	85(3.5)	
**Infant PMTCT intervention**				
NVP/CTX/AZT	60 (2.5)	2,313 (97.5)	2,373 (98.7)	<0.001^f^
None	2 (13.3)	13 (86.7)	15 (0.6)	
Unknown	6 (35.3)	11 (64.7)	17 (0.7)	
**Clinical outcome**				
Alive on HAART	51 (100.0)	0 (0.0)	51 (2.1)	<0.001^f^
Dead	7 (100.0)	0 (0.0)	7 (0.3)	
Discharged^i^	0 (0.0)	2,335 (100.0)	2,335 (97.1)	
Lost to follow up	4 (80.0)	1 (20.0)	5 (0.2)	
Transferred out^i^^,^^j^	6 (85.7)	1 (14.3)	7 (0.3)	

HEI: HIV-exposed infant; ANC: antenatal care; ART: anti-retroviral therapy; ARV: anti-retroviral drugs; CD4: Cluster differentiated cell type 4; NVP: nevirapine; PMTCT: prevention of mother-to-child transmission of HIV; CTX: cotrimoxazole; AZT: azidothymidine; ART: anti-retroviral therapy; DNA: Deoxyribonucleic acid; PCR: Polymerase chain reaction.

* Row percentages.

** Column percentages.

^a^ As at time of antenatal care booking for pregnancy with study infant, unless otherwise indicated.

^b^ Includes single, divorced and widowed women.

^c^ Patients’ homes, churches/religious centers and traditional birth attendant centers.

^d^ Received during antenatal care or labour.

^e^ Earliest DNA PCR test.

^f^ Likelihood ratio.

^g^ Fisher’s exact.

^h^ At antenatal booking for index pregnancy.

^i^ From PMTCT program to regular pediatric care.

^j^ Formal transfer of care to another facility.

### Final HIV status and clinical assessment among HIV-exposed infants

**Fig 1:**
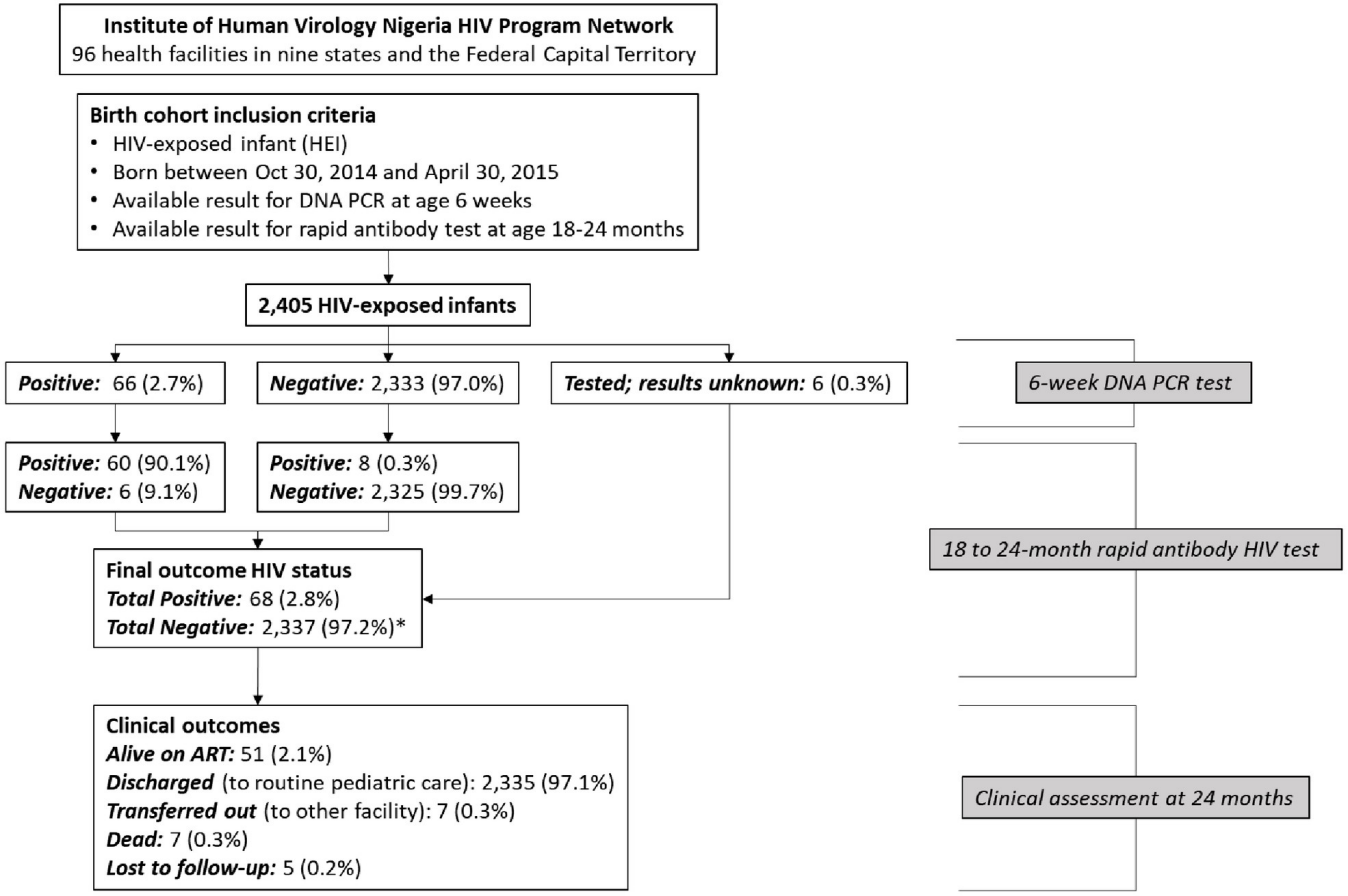
Flow chart for HIV-exposed infants from early diagnosis to final outcome. *Includes 6 infants with unknown 6-week DNA PCR results who subsequently tested negative at 18 to 24-month final outcome assessment.

Out of the 2,405 HEI, 68 (2.8%) had an HIV-positive final outcome status. Of the these, 8 (11.8%) initially tested HIV-negative, and then converted to HIV-positive for final outcome, while 6 (9%) of previously DNA PCR positive HEI tested negative at final outcome (Fig 1). Out of the 68 HEI with HIV positive final outcome, 75% (n = 51) were alive and on anti-retroviral therapy, 10.3% (n = 7) had died, 5.9% (n = 4) were lost to follow-up and 8.8% (n = 6) were transferred out by age 24 months. Furthermore, among HEI with HIV-negative final outcome, 2,335 (99.9%) were discharged to routine pediatric care, 1 (0.04%) was lost to follow-up and 1 (0.04) was transferred out to another facility (Table 2). Clinical assessment also showed that of the 2,405 infants, 51 (2.1%) were alive and on ART, 2,335 (97.1%) were discharged to routine pediatric care, 7 (0.3%) were transferred out to other facility, 7 (0.3%) died and 5 (0.2) were lost to follow-up (Fig 1).

### Relationship between maternal factors and final HIV status of HIV-exposed infants

In bivariate regression analysis, maternal marital status of “married” was associated with infants’ HIV-positive final outcome (3% married vs 0% not married, p = 0.032) (Table 2). Maternal educational level was also significantly associated with HIV-positive final outcomes among HEI, with the proportion positive decreasing as the level of maternal education increased, from 3.1% among women with primary education to 1.8% among those with secondary or post-secondary education (p = 0.007). Interestingly, there were no HIV-positive infants among women with no education in our cohort. Furthermore, maternal unemployment (p = 0.042), rural residence (p = 0.002), CD4 >500 copies/ml (p = 0.004), and non-receipt of ART (p<0.001) were also associated with HIV-positive final outcome among HEI (Table 2).

### Relationship between infant factors and final HIV status of HIV-exposed infants

Bivariate analysis revealed that compared to those whose mothers received PMTCT drug interventions (2.5%), a higher proportion of infants with unknown (35.3%) or no (13.3%) maternal PMTCT intervention were ultimately HIV-positive (p<0.001, Table 2). Infant birthweight <2.5 kg, maternal-reported mixed feeding methods, and as expected, positive DNA PCR at or before 6 weeks of life also correlated with final HIV positive status.

### Predictors of HIV positive final outcomes among HIV-exposed infants

Multivariate logistic regression analysis determined characteristics associated with lower odds of HIV-positive final outcomes as maternal urban residence (OR 0.47, p = 0.006) and exclusive breastfeeding (OR 0.52, p<0.001). Maternal delivery outside health facilities (OR 2.20, p = 0.025), non-receipt of maternal ART (OR 4.76, p = 0.024), or of infant PMTCT interventions (OR 4.57, p = 0.021) and infant birthweight < 2.5kg (OR 0.48, p = 0.022) were associated with higher odds of HIV-positive final outcomes for HEI (Table 3).

**Table 3:** Multivariate logistic regression analysis for predictors of final HIV outcomes of HIV-exposed infants.

Variable	Crude OR [95% CI]	p	Adjusted OR [95% CI]	p
**Maternal ART/ARV prophylaxis** ^a^				
Yes	1.0 [Ref]		1.0 [Ref]	
No	9.59 [3.15–29.19]	**<0.001**	4.76 [1.23–18.42]	**0.024**
Unknown	5.69 [3.25–9.96]	**<0.001**	8.14 [3.78–17.55]	**<0.001**
**Delivery mode**				
Caesarean section	1.0 [Ref]		1.0 [Ref]	
Spontaneous vaginal	2.41 [0.58–9.93]	0.224	3.23 [0.69–15.15]	0.137
**Maternal place of delivery**				
Health facility	1.0 [Ref]		1.0 [Ref]	
Non-health facility^b^	2.20 [1.10–4.39]	**0.025**	1.28 [0.51–3.22]	0.597
Unknown	0.60 [0.08–4.38]	0.611	0.15 [0.02–1.46]	0.102
**Maternal residence**				
Rural	1.0 [Ref]		1.0 [Ref]	
Urban	0.47 [0.28–0.81]	**0.006**	0.45 [0.25–0.81]	**0.008**
Unknown	1.45 [0.67–3.11]	0.343	0.29 [0.11–0.79]	**0.016**
**Infant feeding practice**				
Mixed feeding	1.0 [Ref]		1.0 [Ref]	
Exclusive breast feeding	0.52 [0.30–0.13]	**<0.001**	0.04 [0.02–0.10]	**<0.001**
Exclusive replacement	Omitted		Omitted	
Missing	0.16 [0.05–0.50]	**0.001**	0.06 [0.02–0.24]	**<0.001**
**Infant birth weight**				
<2.5kg	1.0 [Ref]		1.0 [Ref]	
≥2.5kg	0.48 [0.26–0.90]	**0.022**	0.40 [0.21–0.80]	**0.009**
**Infant PMTCT intervention**				
NVP/CTX/AZT	1.0 [Ref]		1.0 [Ref]	
None	4.57 [1.31–26.83]	**0.021**	2.39 [0.43–13.37]	0.320
Unknown	11.02 [7.53–58.73]	**<0.001**	8.88 [1.87–42.07]	**0.006**

ART: anti-retroviral therapy; ARV: anti-retroviral drugs; NVP: Nevirapine; PMTCT: Prevention of mother-to-child transmission of HIV; CTX: cotrimoxazole; AZT: Azidothymidine.

^a^ Received during antenatal care or labour.

^b^ Patients’ homes, churches/religious centers and traditional birth attendant centers.

The full model containing all predictors was statistically significant χ^2^ (12) = 105.52, p<0.001, indicating ability to differentiate HEI with positive or negative outcomes at ≥18 months. The model explained 17% (Pseudo R^2^ = 0.1721) of the variability in HEI outcomes and correctly classified 76.5% of the cases. HEI with birthweight of ≥2.5 kg had 60% (aOR 0.40, p = 0.009) reduced risk of being HIV-positive compared to those with low birthweight of <2.5 kg. Pregnant women living with HIV in urban areas had a 55% (aOR 0.45, p = 0.008) decreased likelihood of having an infant with final HIV positive status compared to those living in rural areas. HEI who were reported as exclusively breastfed for six months had a 96% reduced likelihood of vertical transmission (aOR 0.04, p<0.001) compared to those receiving mixed feeding. Exclusive replacement feeding of HEI perfectly predicted negative HIV status of HEI at 18 months, which was why it was omitted from the model.

Pregnant women living with HIV who did not receive ART antenatally or during labour were nearly 10 times (aOR 9.59, p<0.001) more likely to transmit HIV to their infants compared to women who received chemoprophylaxis.

## Discussion

In this retrospective cross-sectional study, 68 out of 2,405 (2.8%) HIV-exposed infants tested HIV-positive at final outcome assessment in Nigeria. This is lower than the national estimate of 25% [1], but higher than post-breastfeeding/final outcome rates of 0.7%-1% available from prior Nigerian studies using data collected between 2010 and 2016 in the North-Central [14, 19] and South-East zones [20]. Compared to the 2020 national estimates of 15% and 10% respectively [1], the relatively low early (2.7%) and final (2.8%) MTCT rates in our infant cohort, is encouraging. However, this data is not population-based and as such, may underestimate final outcome MTCT rates. Furthermore, our analysis is based on 2016 final outcomes data, and may not reflect the current situation.

Nonetheless, available data on final outcomes, including population-based data in African countries is scarce. A population-based South African study reported an early (4 to 8-week) postpartum MTCT rate of 3.5% among 10,178 HEI; however, there was no final outcome analysis or data reported [21]. A study from Zimbabwe reported an 18-month MTCT final outcome of 0.3% among a nationally-representative sample of 6,051 HEI [22], which was approximately 10 times lower than we found in our Nigerian cohort. However, there is wide variation across the continent; non-population-based studies from other African countries have reported post-breastfeeding/final outcome HIV positivity rates ranging from 2.2% to 9.6% for cohorts with approximately 170 to 3,780 HEI [23–27].

Our final outcome analysis showed that 0.3% (n = 8) of 2,333 HEI with negative six-week DNA PCR tests were ultimately found HIV-positive at or after 18 months of age. HEI who initially test PCR-negative can later become HIV-infected due to late *ex utero* MTCT (e.g. during breastfeeding or mixed feeding). Also, approximately 10% of previously PCR-positive infants tested negative at final outcome assessment. The initial PCR positive results may have represented false positives due to the presence of proviral HIV DNA or cell associated HIV RNA that increase the sensitivity of EID assays [28]; or cross-contamination of samples in the laboratory, which may be less likely [29].

### Maternal ART/ARV prophylaxis

In our study, lack of, or unknown receipt of maternal ART, which also served as infant MTCT prophylaxis, was associated with HIV-positive final outcome status among HEI. This is a well-documented phenomenon among both final outcome [19, 22] and early MTCT studies [21, 30]. Prior studies have established that lack of; or inadequate HIV treatment or prophylaxis results in a higher maternal viral load during pregnancy, delivery or breastfeeding [31–33].

The uptake of maternal ART/ARV (antiretrovirals) in our study (91.6%) is higher than the 2020 coverage (44%) for eligible pregnant women in Nigeria’s PMTCT program [1]. This should be interpreted with caution, as our study is not population-based and did not include pregnant women who did not enroll in, or have access to the services and support received in PMTCT programs in the nine included states or across the entire country. Several factors occurring throughout the PMTCT cascade including at the interpersonal, community, healthcare facility and policy/governance levels contribute to low access to maternal ART in Nigeria and other African countries [34–40]. We expect that as lifelong treatment coverage improves in Nigeria, more women living with HIV would have early access, remain adherent and on ART throughout their first and subsequent pregnancies. Also, increasingly accessible point-of-care and birth testing strategies would enable prompt linkage of infants testing HIV-positive to lifesaving ART and minimize missed opportunities for presentation, testing and linkage.

### Maternal residence

Pregnant women living with HIV in rural areas were about two times more likely to transmit HIV to their HEIs compared to their urban counterparts, similar to findings in Ethiopia [41]. Another study posited that urban-dwelling women have better knowledge about HIV than their rural counterparts, due in part to less access to education campaigns in poorly-reached areas [42]. Among urban women, easier access to health facilities and skilled healthcare workers contribute to lower rates of HIV positive final outcomes among HEI, due to increased receipt of services like adherence counselling and quality patient-provider engagements; while an underdeveloped transport system, poor telecommunications, drug stock outs, understaffed and over pressured primary health centers, and inadequately trained clinic staff typify the structural barriers to PMTCT in rural areas [43–45].

### Infant feeding practice

We reported a higher proportion and increased likelihood of HIV positive final outcomes for HEI who received mixed feeding compared to those on exclusive breastfeeding. Exclusive breastfeeding protects against HIV transmission by protecting the integrity of the mucosa through antibodies and innate factors in breast milk which inhibit the uptake, transport, and replication of HIV-1 [46]. Mixed feeding with infant formula causes non-breast milk antigen-induced inflammation that increases infants’ vulnerability to HIV infection [47]. Compared to mixed feeding, exclusive breastfeeding is less liable to cause breast health issues like subclinical mastitis and breast abscesses which are associated with increased breastmilk viral loads [48]. Ultimately, infants receiving mixed feeding are at as much as 42 times higher risk of acquiring HIV infection compared to those exclusively breastfeeding [41].

Although we did not explore these or similar factors, among mothers living with HIV, lack of formal education [49] lack of pre-existing norms for exclusive breast feeding, financial vulnerability, non-disclosure of HIV status, perception of insufficient breast milk, social and family advice or pressure/influence, and conflicting messages from healthcare workers have been associated with mixed feeding, which increases the risk of MTCT [50].

### Infant birthweight

Several studies have established an association between maternal HIV infection and an increased risk of infant low birth weight [51, 52]. In our study, babies with low birth weight of ≤2.5 kg had a higher risk of HIV-positive outcomes than those with birth weight >2.5 kg, similar to prior findings in Zimbabwe [53]. Although the mechanism of the interaction between maternal HIV and low infant birth weight is still unknown, a speculated cause is HIV infection-induced placental inflammation that disrupts placental functions, including maternal-fetal exchange [54].

### Infant PMTCT intervention

Similar to previous studies in Ethiopia and Nigeria [26, 55, 56], infants who did not receive ARV prophylaxis, or for whom this was unknown were more likely to be HIV-positive at 18 months compared to those who received ARV prophylaxis. Infant ARV prophylaxis reduces the risk of MTCT during pregnancy, labour, delivery, and breastfeeding [26].

### Factors not associated with MTCT (non-findings)

Although there was no statistical difference in final infant outcomes for mothers who delivered at or outside of health facilities in our study, other studies have reported that delivery in non-health facilities is associated with poor outcomes for infants of pregnant women living with HIV [14, 25, 41], due to lack of skilled birth attendants to ensure safe delivery practices, and the unavailability of PMTCT interventions typically available at health facilities [26]. Several studies have reported that spontaneous vaginal delivery compared with assisted delivery [26] or elective caesarean section compared to vaginal delivery [20, 32] is associated with negative final infant HIV outcomes, which our study was not designed to evaluate.

### Limitations

Our study is limited by variables missing/unknown, some of which we are unable to explain. These missing data include two month EID information, as only six week EID data were available for detailed analysis. We did not apply further statistical approaches—e.g. multiple imputation- to address missing data, which can generate misleading results; we found our simpler approach sufficient for our purpose. Furthermore, we were unable to classify DNA PCR result by infant chronological age, due to incomplete records for mother-infant pairs. The HIV status of HEI who did not present for early and/or final testing was not evaluated. Also, HEI presenting later than six weeks for EID and earlier than 18 months for final outcomes were excluded from analysis. This done was to reduce confounding of outcomes assessments related to programmatically-recommended testing time-points for EID and final outcome. It is plausible that the HIV positivity rate would likely have been higher if data from “off-schedule” infant testing were included. The absence of maternal viral load data limits analysis of its role as a predictor of HEI final outcome in this study. Finally, the retrospective collection of data might have been affected by data collection errors.

### Strengths of the study

The analysis of a birth cohort of more than 2,400 HEI-maternal pairs across 10 of Nigeria’s 37 states and territories improved the study’s reliability and validity. Furthermore, data was collected from both public and private health facilities across all healthcare delivery levels in both rural and urban settings in the included states, strengthening study generalizability. Finally, the application of multivariate analysis to routinely reported programmatic data addresses potential cofounders to better reflect real-world outcomes and impact of the PMTCT program.

## Conclusions

Our study assessed 18 to 24 month final outcomes for a relatively large cohort of HIV-exposed infants in Nigeria’s PMTCT program. While this analysis was not population based, it provides further insight into downstream impact of maternal-level interventions on infants and highlights factors associated with a final infant HIV positive outcome, essentially a failure of the PMTCT program.

Drawing from our findings, we recommend strengthened programmatic focus on early access to quality antenatal care and maternal ART, especially for women living with HIV in rural areas. Furthermore, we recommend strengthening and sustaining nationwide implementation of education and sensitization on six-months of exclusive infant breastfeeding concurrent with maternal ART. As maternal ART regimens change and coverage improves, timely, population-level data on HEI final outcome is needed to further inform PMTCT policy and implementation.
